# Correlation of French Federation of Cancer Centres Sarcoma Group (FNCLCC) Grade With Ki-67 Proliferation Index in Malignant Soft Tissue Sarcomas

**DOI:** 10.7759/cureus.111699

**Published:** 2026-06-29

**Authors:** Ruchi Yadav, Mala Sagar, Malti Kumari, Madhu Kumar, Babli

**Affiliations:** 1 Department of Pathology, Maharishi Chyawan Government Medical College, Mahendragarh, IND; 2 Department of Pathology, King George's Medical University, Lucknow, IND; 3 Department of Pathology, Pandit Neki Ram Sharma Government Medical College, Bhiwani, IND

**Keywords:** clinical skills, competency-based medical education, direct observation of procedural skills, internship training, workplace-based assessment

## Abstract

Background: Soft tissue sarcomas are rare malignant tumours comprising diverse histological subtypes with variable biological behaviour. Histological grading using the French Federation of Cancer Centres Sarcoma Group (FNCLCC) system is widely applied for prognostic stratification. Immunohistochemical advances have introduced proliferative markers such as Ki-67 as potential adjuncts to conventional grading.

Objective: This study aimed to determine the correlation between the Ki-67 proliferation index and histological grade of soft tissue sarcomas using the FNCLCC grading system.

Methods: This retrospective observational study included 49 histologically confirmed malignant soft tissue sarcomas evaluated at a tertiary care centre over a one-year period. Eligible incisional and excisional biopsy specimens were assessed histopathologically and graded using the FNCLCC grading system. Ki-67 immunohistochemistry was performed, and the proliferation index was categorised as low, intermediate, or high. The association between FNCLCC grade and Ki-67 proliferation index was analysed using the chi-square test, with p < 0.05 considered statistically significant.

Results: The mean age was 30.6 years with male predominance (male: female = 1.7:1). The lower extremity was the most common site, and Ewing’s sarcoma was the predominant subtype (22.4%). Tumours were classified as Grade 1 (14.3%), Grade 2 (42.9%), and Grade 3 (42.9%). A significant association was observed between FNCLCC grade and Ki-67 proliferation index (p < 0.001).

Conclusion: The Ki-67 proliferation index showed a significant correlation with FNCLCC histological grade in malignant soft tissue sarcomas and may be considered an adjunctive histopathological marker of proliferative activity. However, larger prospective studies with subtype-stratified analysis and clinical outcome correlation are necessary to determine its independent prognostic relevance and practical applicability.

## Introduction

Soft tissue sarcomas are rare malignant neoplasms of mesenchymal origin, with an annual incidence of approximately 30 cases per million people and accounting for less than 1% of all malignant tumours [1,2]. They comprise a heterogeneous group of tumours that may show adipocytic, fibrohistiocytic, smooth muscle, skeletal muscle, vascular, or peripheral nerve sheath differentiation, each with distinct morphological and biological characteristics [1,3]. This heterogeneity creates diagnostic challenges in routine histopathological practice, particularly in small biopsies and poorly differentiated lesions, where overlapping morphology and variable differentiation may complicate classification and prognostic assessment [4].

Histological grading is, therefore, an important component of soft tissue sarcoma evaluation, as it helps estimate tumour aggressiveness and clinical behaviour [5,6]. Although histological subtype remains a key determinant of outcome, grading provides additional prognostic stratification by incorporating features that reflect biological activity [5]. Several grading systems have been proposed since the first formal grading approach for soft tissue sarcomas was introduced in 1977 [5,6]. Among these, the French Federation of Cancer Centres Sarcoma Group (FNCLCC) grading system is the most widely used and clinically applicable model [7].

The FNCLCC system has been validated through comparative and outcome-based studies [7,8]. It assesses three parameters: tumour differentiation, mitotic count, and tumour necrosis, each assigned a numerical score [8]. The combined score classifies tumours as Grade 1, Grade 2, or Grade 3, corresponding to increasing metastatic potential and poorer prognosis [7,9]. Its reproducibility has also been evaluated in multi-observer studies, supporting its use in routine diagnostic practice [10].

Despite its established role, FNCLCC grading has limitations. Mitotic count, a major grading component, is subject to interobserver variability and may be influenced by tumour heterogeneity, sampling variation, field selection, fixation quality, and section thickness [10]. These factors may affect final grade assignment, particularly in limited biopsy specimens. This has encouraged the use of more objective and reproducible markers of cellular proliferation as adjuncts to conventional histological assessment.

Ki-67 is a well-established immunohistochemical marker of cellular proliferation [11]. It is expressed during active phases of the cell cycle, including G1, S, G2, and M phases, but is absent in the quiescent G0 phase, making it a useful indicator of tumour growth fraction [12]. Ki-67 has been widely used as a proliferation marker in solid tumours and may provide additional biological information beyond routine morphology [13].

In soft tissue sarcomas, proliferative activity is closely related to tumour aggressiveness and clinical outcome [5,14]. Previous studies have reported associations between Ki-67 labelling index, mitotic activity, tumour cellularity, and histological grade in soft tissue sarcomas [15,16]. These findings suggest that Ki-67 may reflect intrinsic tumour biology and complement established grading systems, particularly when mitotic assessment is limited by sampling or interpretive variability.

Assessing the relationship between Ki-67 proliferation index and FNCLCC grade may help clarify the adjunctive value of Ki-67 in soft tissue sarcoma evaluation. The present study, therefore, aimed to determine the association between Ki-67 proliferation index and FNCLCC histological grade in malignant soft tissue sarcomas.

Objectives of the study

This study aimed to evaluate the association between Ki-67 proliferation index and FNCLCC histological grade in malignant soft tissue sarcomas and to assess its adjunctive value as a histopathological marker of proliferative activity within the limits of conventional grading.

## Materials and methods

Study design and sample selection

This retrospective observational study was conducted at King George’s Medical University, a tertiary care centre in Lucknow, India, over a one-year period. All histologically confirmed malignant soft tissue sarcomas identified during the study period were screened for inclusion. Demographic and clinicopathological data, including age, sex, tumour site, biopsy type, and histopathological diagnosis, were retrieved from patient records and departmental archives. Institutional ethics approval was obtained before data collection, as per institutional requirements.

Adequately sized incisional biopsy specimens measuring more than 2 cm, and all excisional biopsy specimens were included. Trucut biopsies and incisional biopsy specimens measuring less than 2 cm were excluded because small tissue samples may not adequately represent tumour heterogeneity, necrosis, mitotic activity, and differentiation, all of which are required for reliable FNCLCC grading. Limited sampling may also affect Ki-67 assessment by underrepresenting areas of highest proliferative activity. Poorly preserved tissue samples unsuitable for histopathological or immunohistochemical evaluation were also excluded. Solitary fibrous tumour and malignant peripheral nerve sheath tumour were excluded because FNCLCC grading is not uniformly recommended for these tumour types and remains subject to diagnostic and prognostic debate in the literature [7].

Histopathological evaluation

To preserve optimal morphological detail, tissue specimens were fixed in 10% neutral buffered formalin immediately after receipt. After fixation, tissues were routinely processed, dehydrated, cleared, and embedded in paraffin blocks according to standard laboratory procedures. Sections measuring 3-5 µm were cut from paraffin-embedded blocks and stained with hematoxylin and eosin for histopathological examination.

Microscopic evaluation was performed to identify the histological subtype based on morphological features. Immunohistochemical markers were used when required to support tumour typing and lineage differentiation. All cases were assessed independently by two pathologists who were blinded to the clinical details. Tumours were graded using the FNCLCC grading system. The scoring parameters included tumour differentiation, mitotic count, and percentage of tumour necrosis. Each parameter was assigned a numerical score, and the combined score was used to classify tumours as Grade 1, Grade 2, or Grade 3. The grading criteria are summarised in Table 1.

**Table 1:** FNCLCC grading system Modified from Trojani et al. [8]; the FNCLCC grading system is a literature-based, non-proprietary tool. FNCLCC: French Federation of Cancer Centres Sarcoma Group; PNET: primitive neuroectodermal tumour, HPF: high power field *An HPF measures 0.1734 mm²

Component	Assigned Score	Diagnostic Criteria
Tumor differentiation	1	Tumours closely resembling normal mature mesenchymal tissue (for example, low-grade leiomyosarcoma).
	2	Tumours with definite histological classification (for example, myxoid liposarcoma).
	3	Poorly differentiated or embryonal sarcomas, tumours of uncertain lineage, synovial sarcoma, osteosarcoma, and PNET.
Mitotic activity (per 10 HPF*)	1	0–9 mitotic figures
	2	10–19 mitotic figures
	3	20 or more mitotic figures
Tumor necrosis	0	Absence of necrosis
	1	Necrosis involving less than 50% of the tumour area
	2	Necrosis involving 50% or more of the tumour area
Final histological grade	Grade 1	Combined score of 2–3
	Grade 2	Combined score of 4–5
	Grade 3	Combined score of 6–8

Immunohistochemical analysis

Ki-67 immunohistochemistry was performed to assess proliferative activity. Representative paraffin blocks were sectioned at 3-4 µm thickness and mounted on 3-aminopropyltriethoxysilane-coated slides to improve tissue adhesion. Ki-67 immunostaining was performed on the Dako Omnis platform (Agilent Technologies, Santa Clara, CA, USA) using the monoclonal anti-Ki-67 antibody, clone MIB-1 (Dako/Agilent Technologies, Santa Clara, CA, USA), according to standard immunohistochemical protocols. Appropriate controls were included in each staining batch. Human tonsil tissue was used as the positive control because of its known high proliferative activity, while negative controls were prepared by omitting the primary antibody to confirm staining specificity.

Stained slides were examined at ×400 magnification. Tumour cells showing diffuse granular or globular nuclear staining were considered positive for Ki-67, irrespective of staining intensity. Cytoplasmic staining was not included in the assessment. Areas showing the highest density of Ki-67-positive tumour cells were identified at low magnification, and approximately 500 tumour cells were evaluated across 10 high-power fields in these selected regions [11-13]. Ki-67 scoring was performed independently by two pathologists blinded to clinical details, and discrepant assessments were reviewed jointly to reach consensus.

The Ki-67 proliferation index was calculated as the percentage of tumour cells showing positive nuclear staining among the total number of tumour cells counted. Cases were categorised into three groups: low index (<25 positive cells/10 high-power fields), intermediate index (25-50 positive cells/10 high-power fields), and high index (>50 positive cells/10 high-power fields). These cut-offs were used as pragmatic analytical thresholds to enable comparison with FNCLCC grade categories and were informed by the observed distribution of Ki-67 expression in the present cohort as well as prior studies evaluating Ki-67 labelling index in soft tissue sarcomas [15,17]. As no universally accepted Ki-67 cut-off exists for grading all soft tissue sarcoma subtypes, these categories were interpreted as adjunctive analytical groups rather than independently validated prognostic thresholds.

Statistical analysis

SPSS software, version 15.0 (SPSS Inc., Chicago, IL, USA), was used to tabulate and analyse the collected data. Continuous variables were summarised using mean and standard deviation, while categorical variables were summarised using frequencies and percentages. The association between FNCLCC grade and Ki-67 proliferation index categories was evaluated using the chi-square test. Fisher’s exact test was applied for contingency tables with small expected cell counts. A p-value of less than 0.05 was considered statistically significant for all analyses.

## Results

Clinicodemographic characteristics

The study included 49 histologically confirmed malignant soft tissue sarcomas, comprising 10 incisional biopsies and 39 excisional biopsies. Patient age ranged from 14 months to 97 years, with a mean age of 30.6 years. Most patients were younger than 40 years, and males predominated with a male-to-female ratio of 1.7:1. The clinicodemographic profile is summarised in Table 2.

**Table 2:** Clinicodemographic characteristics of the study population (n=49)

Variable	Category	Number of patients	Percentage (%)
Biopsy type	Incisional	10	20.4
	Excisional	39	79.6
Age group (years)	<20	22	44.9
	21-40	16	32.6
	41-60	06	12.2
	>60	05	10.2
Gender distribution	Male	31	63.3
	Female	18	36.7

Histopathological diagnosis and tumour site distribution

The lower extremity was the most frequently involved anatomical site, followed by the trunk and upper extremity. Ewing sarcoma was the most common histopathological subtype, followed by synovial sarcoma and rhabdomyosarcoma. Several subtypes, including rhabdomyosarcoma, Ewing sarcoma, synovial sarcoma, and angiosarcoma, were observed predominantly in younger patients. The distribution of histological subtypes and tumour sites is presented in Table 3.

**Table 3:** Histopathological diagnosis and tumour site distribution (n = 49)

Variable	Category	Number of patients	Percentage (%)
Histopathological diagnosis	Rhabdomyosarcoma	08	16.3
	Ewing’s sarcoma	11	22.4
	Mesenchymal chondrosarcoma	02	4.1
	Fibrosarcoma	06	12.2
	Synovial sarcoma	10	20.4
	Undifferentiated pleomorphic sarcoma	04	8.2
	Leiomyosarcoma	03	6.1
	Liposarcoma	03	6.1
	Angiosarcoma	01	2.0
	Alveolar soft part sarcoma	01	2.0
Site	Back	03	6.1
	Head and Neck	06	12.2
	Lower extremity	15	30.6
	Mesentry	03	6.1
	Pelvis	04	8.2
	Retroperitoneum	03	6.1
	Trunk	08	16.3
	Upper extremity	07	14.3

FNCLCC grading distribution

All tumours were graded using the FNCLCC histological grading system. Among incisional biopsy specimens, no Grade 1 tumours were identified; Grade 2 and Grade 3 tumours each accounted for five cases. A significant association was observed between histological subtype and FNCLCC grade in incisional biopsies (χ² = 10.000, df = 3, p = 0.019), with Ewing sarcoma representing all Grade 3 cases in this group. The detailed distribution is shown in Table 4.

**Table 4:** Grading of incisional biopsy as per the FNCLCC grading system (n = 10) χ2 = 10.000 (df = 3), p = 0.019 (significant); Grading based on the FNCLCC system [7,8] FNCLCC: French Federation of Cancer Centres Sarcoma Group

Diagnosis	Total (n)	Grade 1 (n)	Grade 1 (%)	Grade 2 (n)	Grade 2 (%)	Grade 3 (n)	Grade 3 (%)
Rhabdomyosarcoma	1	0	0.0	1	100.0	0	0.0
Ewing’s sarcoma	5	0	0.0	0	0.0	5	100.0
Synovial sarcoma	3	0	0.0	3	100.0	0	0.0
Alveolar soft part sarcoma	1	0	0.0	1	100.0	0	0.0
Total	10	0	0.0	5	50.0	5	50.0

Among excisional biopsy specimens, Grade 1 tumours accounted for seven cases, while Grade 2 and Grade 3 tumours each accounted for 16 cases. A significant association was also observed between histological subtype and FNCLCC grade in excisional biopsies (χ² = 50.563, df = 16, p < 0.001). Lower-grade tumours were more frequently represented by liposarcoma, leiomyosarcoma, and fibrosarcoma, whereas Ewing sarcoma and undifferentiated pleomorphic sarcoma were consistently classified as Grade 3. The distribution is summarised in Table 5.

**Table 5:** Grading of excisional biopsy as per the FNCLCC grading system (n = 39) χ2 = 50.563 (df = 16); p < 0.001 (Significant); Grading based on the FNCLCC system [7,8] FNCLCC: French Federation of Cancer Centres Sarcoma Group

Diagnosis	Total (n)	Grade 1 (n)	Grade 1 (%)	Grade 2 (n)	Grade 2 (%)	Grade 3 (n)	Grade 3 (%)
Rhabdomyosarcoma	7	0	0.0	3	42.9	4	57.1
Ewing’s sarcoma	6	0	0.0	0	0.0	6	100.0
Mesenchymal chondrosarcoma	2	0	0.0	2	100.0	0	0.0
Fibrosarcoma	6	2	33.3	4	66.7	0	0.0
Synovial sarcoma	7	0	0.0	6	85.7	1	14.3
Undifferentiated pleomorphic sarcoma	4	0	0.0	0	0.0	4	100.0
Leiomyosarcoma	3	2	66.7	0	0.0	1	33.3
Liposarcoma	3	3	100.0	0	0.0	0	0.0
Angiosarcoma	1	0	0.0	1	100.0	0	0.0
Total	39	7	17.9	16	41.0	16	41.0

Correlation between the FNCLCC grade and Ki-67 proliferation index

Ki-67 proliferation index showed a clear positive association with FNCLCC grade in both incisional and excisional biopsy specimens. In incisional biopsies, all Grade 2 tumours showed a low Ki-67 index, whereas most Grade 3 tumours showed a high Ki-67 index. This association was statistically significant (χ² = 10.000, df = 2, p = 0.007).

A similar pattern was observed in excisional biopsies. All Grade 1 and Grade 2 tumours showed a low Ki-67 index, while most Grade 3 tumours showed a high Ki-67 index, with the remaining Grade 3 cases showing an intermediate index. The association between increasing FNCLCC grade and higher Ki-67 proliferation index was statistically significant (χ² = 39.000, df = 4, p < 0.001). These findings demonstrate an association between FNCLCC histological grade and Ki-67 proliferation index categories in this cohort, as shown in Table 6.

**Table 6:** Correlation of FNCLCC grading with Ki-67 index according to type of biopsy Incisional biopsy: χ² = 10.000 (df = 2), p = 0.007 (significant); Excisional biopsy: χ² = 39.000 (df = 4), p < 0.001 (significant) Ki-67 proliferation index assessment and categorisation were performed using standard immunohistochemical evaluation methods supported by previous studies [11–13,15]. FNCLCC: French Federation of Cancer Centres Sarcoma Group

Type of Biopsy	FNCLCC Grade	Total (n)	0–25 (n)	0–25 (%)	26–50 (n)	26–50 (%)	>50 (n)	>50 (%)
Incisional	Grade I	0	0	0.0	0	0.0	0	0.0
	Grade II	5	5	100.0	0	0.0	0	0.0
	Grade III	5	0	0.0	1	20.0	4	80.0
	Total	10	5	100.0	1	100.0	4	100.0
Excisional	Grade I	7	7	100.0	0	0.0	0	0.0
	Grade II	16	16	100.0	0	0.0	0	0.0
	Grade III	16	0	0.0	4	25.0	12	75.0
	Total	39	23	100.0	4	100.0	12	100.0

**Figure 1:**
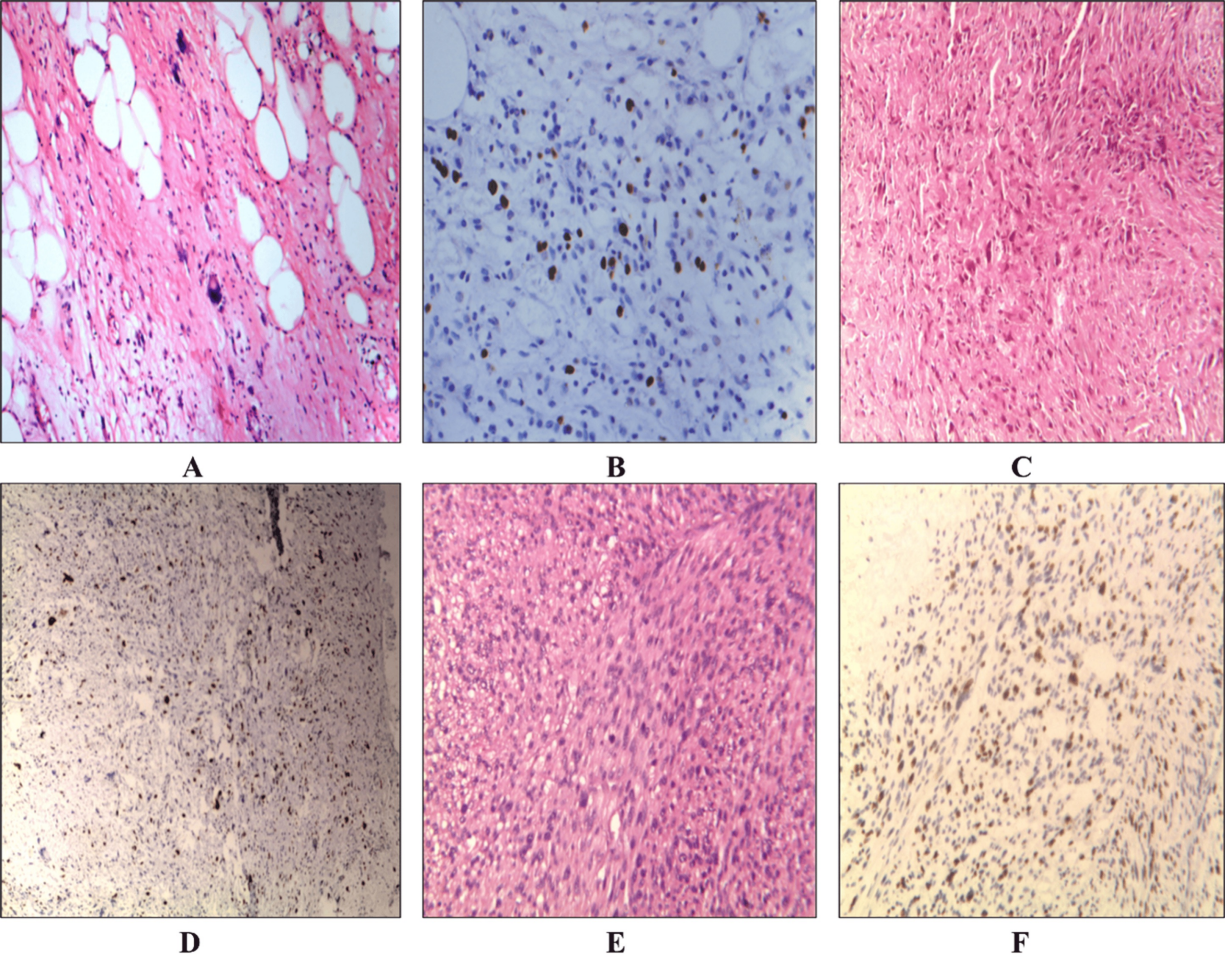
H&E sections and Ki-67 index across proliferation groups A. Liposarcoma: H&E (×400); B. Corresponding low Ki-67 PI (×100); C. Undifferentiated pleomorphic sarcoma: H&E (×100); D. Corresponding intermediate Ki-67 PI (×100), E. Leiomyosarcoma: H&E (×100); F. Corresponding high Ki-67 PI (×100). Ki-67 immunohistochemical staining and proliferation index assessment were performed using standard methods as described in the literature [11–13].

The representative histological and immunohistochemical staining patterns across Ki-67 proliferation groups are illustrated in Figure 1.

## Discussion

The present study evaluated the association between FNCLCC histological grade and Ki-67 proliferation index in malignant soft tissue sarcomas and demonstrated a statistically significant positive relationship between increasing histological grade and higher proliferative activity. The cohort showed a wide age distribution, with a predominance of younger patients, particularly those below 20 years of age. Male predominance and lower-extremity involvement were also observed. These demographic and site-related findings are broadly comparable with previously published Indian institutional series reporting male predominance and frequent extremity involvement in soft tissue sarcomas [16-18]. In contrast, data from the National Cancer Database in the United States have shown an older age distribution at diagnosis, suggesting possible regional, referral-related, and population-level differences in case composition [19].

Ewing sarcoma was the most common histological subtype in the present cohort, accounting for 22.4% of cases. Rhabdomyosarcoma, Ewing sarcoma, synovial sarcoma, and angiosarcoma were more frequently observed in younger patients, consistent with the known age-related distribution of several sarcoma subtypes. Differences in subtype distribution across studies may reflect institutional referral patterns, geographic variation, sample size, inclusion criteria, and the extent of ancillary diagnostic workup. Older studies have also reported diagnostic categories such as malignant fibrous histiocytoma, which is now largely classified as undifferentiated pleomorphic sarcoma or reclassified into more specific entities when adequate sampling and immunohistochemistry are available.

An important interpretive issue in the present cohort is the predominance of younger patients and high-grade sarcoma subtypes such as Ewing sarcoma and rhabdomyosarcoma. These tumour types are generally assigned a high tumour differentiation score within the FNCLCC system, which can strongly influence the final histological grade. They are also biologically high-proliferation tumours and may therefore show higher Ki-67 expression. As a result, the observed association between Ki-67 proliferation index and FNCLCC grade may partly reflect histological subtype distribution rather than an entirely independent relationship between Ki-67 and tumour grade. This subtype-related confounding is particularly relevant in a small heterogeneous cohort and should be considered when interpreting the strength of the Ki-67/FNCLCC correlation. Larger studies with subtype-stratified or multivariable analysis are required to determine whether Ki-67 provides independent grading or prognostic information beyond histological subtype and FNCLCC parameters.

A significant association was observed between histological subtype and FNCLCC grade in both incisional and excisional biopsy specimens. Biologically aggressive subtypes, including Ewing sarcoma and rhabdomyosarcoma, were more frequently classified as higher grade, whereas more differentiated tumours were more commonly represented in lower-grade categories. This pattern is consistent with the expected biological behaviour of these tumour types. However, because histological subtype contributes directly to tumour differentiation scoring in the FNCLCC system, subtype composition may have influenced both FNCLCC grade distribution and Ki-67 expression patterns. Therefore, the observed Ki-67/FNCLCC relationship should be interpreted with attention to possible subtype-related confounding.

The Ki-67 proliferation index showed a positive association with FNCLCC grade. Low-grade and intermediate-grade tumours generally demonstrated low Ki-67 expression, whereas high-grade tumours more frequently showed high Ki-67 expression. This finding is biologically plausible because Ki-67 reflects the tumour growth fraction and is closely related to cellular proliferation, which overlaps conceptually with the mitotic activity component of FNCLCC grading [11,12]. Previous studies have also reported correlations between Ki-67 labelling index, mitotic activity, tumour cellularity, and histological grade in soft tissue tumours [15,16,20].

Recent studies further support the relevance of Ki-67 as an adjunct marker in soft tissue sarcoma evaluation. Associations between Ki-67 index, FNCLCC grade, and imaging features of tumour aggressiveness have been reported, suggesting that proliferative markers may contribute to broader risk assessment [21]. A significant relationship between the Ki-67 labelling index and FNCLCC grade has also been shown in non-small round cell soft tissue sarcomas [22]. Other studies have indicated that Ki-67 expression and histological grade may have prognostic relevance in adult soft tissue sarcomas [23]. The present findings are consistent with this body of evidence, although the current study was not designed to establish independent prognostic value.

The potential value of Ki-67 lies in its ability to provide an adjunctive estimate of proliferative activity when interpreted with conventional histological grading parameters. However, Ki-67 should not be interpreted as a replacement for FNCLCC grading. Rather, it may serve as an adjunctive marker when integrated with morphology, tumour subtype, mitotic activity, necrosis, and clinical context. Because Trucut biopsies and small incisional biopsy specimens were excluded from the present study, these findings cannot determine the utility of Ki-67 in limited biopsy specimens.

This study has several limitations. The sample size was relatively small, reflecting the rarity of malignant soft tissue sarcomas, and the single-centre design may limit generalisability. The cohort included heterogeneous histological subtypes, including subtypes that tend to receive high FNCLCC differentiation scores and show high proliferative activity, which may have inflated the apparent strength of the Ki-67/FNCLCC association. Survival data, recurrence data, metastasis status, treatment response, and long-term follow-up were not available; therefore, the prognostic significance of Ki-67 could not be assessed. Molecular and genetic profiling was not performed, which may have limited diagnostic refinement in selected sarcoma subtypes. In addition, the Ki-67 categories used in this study were analytical thresholds informed by cohort distribution and prior literature rather than universally validated prognostic cut-offs. Formal interobserver agreement statistics for FNCLCC grading and Ki-67 scoring were not calculated, which may limit assessment of scoring reproducibility. Despite these limitations, the statistically significant association between Ki-67 proliferation index and FNCLCC grade supports the potential adjunctive role of Ki-67 in histopathological evaluation of malignant soft tissue sarcomas. Larger prospective studies incorporating molecular classification, subtype-stratified analysis, multivariable modelling, clinical outcome endpoints, and formal reproducibility assessment are needed to define its prognostic and practical utility more precisely.

## Conclusions

A statistically significant association between Ki-67 proliferation index and FNCLCC histological grade was observed in malignant soft tissue sarcomas. Higher FNCLCC grades were associated with progressively increased Ki-67 expression, supporting a positive relationship between proliferative activity and histological grade. These findings suggest that Ki-67 may serve as an adjunctive histopathological marker of proliferative activity when interpreted alongside tumour morphology, histological subtype, mitotic activity, and necrosis. However, this study does not establish the cost-effectiveness of Ki-67, its utility in small biopsy specimens, its independent prognostic value, or its role in clinical decision-making. Owing to the heterogeneous distribution of tumour subtypes, relatively small sample size, and absence of survival, recurrence, metastasis, or treatment-response data, larger prospective studies with subtype-stratified analysis and clinical outcome correlation are required to define its prognostic relevance and practical applicability in soft tissue sarcomas.
